# Variability and predictors of urinary concentrations of organophosphate flame retardant metabolites among pregnant women in Rhode Island

**DOI:** 10.1186/s12940-017-0247-z

**Published:** 2017-04-11

**Authors:** Megan E. Romano, Nicola L. Hawley, Melissa Eliot, Antonia M. Calafat, Nayana K. Jayatilaka, Karl Kelsey, Stephen McGarvey, Maureen G. Phipps, David A. Savitz, Erika F. Werner, Joseph M. Braun

**Affiliations:** 1 Department of Epidemiology, Brown University School of Public Health, Providence, RI USA; 2 Department of Epidemiology, Geisel School of Medicine at Dartmouth, Lebanon, NH USA; 3 Department of Epidemiology (Chronic Diseases), Yale University School of Public Health, New Haven, CT USA; 4 Division of Laboratory Sciences, National Center for Environmental Health, Centers for Disease Control and Prevention, Atlanta, GA USA; 5 Department of Pathology and Laboratory Medicine, Warren Alpert Medical School of Brown University, Providence, RI USA; 6 Department of Anthropology, Brown University, Providence, RI USA; 7 Department of Obstetrics and Gynecology, Warren Alpert Medical School of Brown University, and Women & Infants Hospital of Rhode Island, Providence, RI USA

**Keywords:** Flame retardants, Pregnancy, Urine metabolites

## Abstract

**Background:**

Organophospate flame retardants (PFRs) are chemicals of emerging concern due to restrictions on polybrominated diphenyl ether flame retardant formulations. We describe the occurrence, variability, and predictors of urinary metabolites of PFRs among pregnant women.

**Methods:**

In 2014–2015, 59 women from Providence, RI provided up to 3 spot urine samples during pregnancy (~12, 28, and 35 weeks’ gestation). We created a pooled urine sample per woman and measured nine relevant metabolites in individual and pooled samples. We used linear mixed models to calculate intraclass correlation coefficients (ICCs) across the 3 measurements and to assess sociodemographic and dietary predictors of PFRs.

**Results:**

The median (IQR) of bis-2-chloroethyl phosphate (BCEP), bis(1,3-dichloro-2-propyl) phosphate (BDCPP), and diphenyl phosphate (DPhP), the metabolites most frequently detected, from pooled samples were: 0.31 μg/L (0.17–0.54), 1.18 μg/L (0.64–2.19), 0.93 μg/L (0.72–1.97), respectively. We observed fair to good reproducibility for BCEP (ICC = 0.50), BDCPP (ICC = 0.60), and DPhP (ICC = 0.43), and excellent agreement between the urinary flame retardant metabolite concentrations averaged across pregnancy versus pooled urine sample concentrations for BCEP (ICC = 0.95), BDCPP (ICC = 0.89), and DPhP (ICC = 0.93). Adjusting for pertinent sociodemographic factors and gestational week of urine collection, each 1 kg increase in pre-pregnancy weight was associated with greater BCEP (1.1%; 95% CI: 0.1, 2.1), BDCPP (1.5%; 95% CI: 0.3, 2.7), and DPhP (0.5%; 95% CI: 0.0, 1.1). Dietary factors were generally not associated with urinary flame retardant metabolites.

**Conclusions:**

Urinary concentrations of BCEP, BDCPP, and DPhP were frequently detected among women in this pilot study and had fair reproducibility across pregnancy. Body size may be an important predictor of urinary flame retardant metabolite concentrations.

**Electronic supplementary material:**

The online version of this article (doi:10.1186/s12940-017-0247-z) contains supplementary material, which is available to authorized users.

## Background

To meet state and federal flammability standards, consumer products such as electronics and furniture are often treated with chemical flame retardants [1]. Following the 2004 phase out of specific commercial mixtures of polybrominated diphenyl ether (PBDE) flame retardants due to health and safety concerns, organophosphate flame retardants (PFRs) including triphenyl phosphate (TPhP), tris (1,3-dichloro-2-propyl) phosphate (TDCPP), tris (1-chloro-2-propyl) phosphate, tris (2-chloroethyl) phosphate (TCEP), tri-p- and tri-o-cresylphosphate, tributyl phosphate (TBuP), tribenzyl phosphate, and novel brominated flame retardants such as 2-ethylhexyl-2,3,4,5-tetrabromobenzoate, have been increasingly used in consumer products including residential furniture and baby products [1–3]. TPhP, TBuP, and tricresylphosphate are also commonly used as plasticizers or lubricants [3–7].

PFRs have been frequently detected in household dust, foam furniture, and other foam products [1, 2, 8–12], and their metabolites have been identified in the urine of members of the general population of the United States (US) and elsewhere [10–18]. However, data on both human exposure to these chemicals and associated potential adverse health outcomes is still limited, particularly during the sensitive window of pregnancy. Preliminary evidence from epidemiological studies supports a role for PFRs in disruption of thyroid hormones [19–21] and sex hormones among men [20], suggesting that PFRs may have adverse influences on endogenous hormones or hormonally mediated endpoints. In addition, TDCPP is a carcinogen included in the State of California’s Proposition 65 list of chemicals known to cause cancer [22]. A small number of prior studies have assessed urinary concentrations of PFR metabolites among maternal-toddler pairs [13, 23, 24], infants [12], or pregnant women [14, 25]. These studies suggest that pregnant women and young children in the US general population are likely to have detectable concentrations of two urinary metabolites of PFRs, bis(1,3-dicholoro-2-propyl) phosphate (BDCPP) and diphenyl phosphate (DPhP) [12–14], and that such metabolites may be variable over the course of pregnancy [14, 25]. The extent of exposures among mothers and young children remains largely unknown and sociodemographic and dietary predictors of urinary concentrations of PFR metabolites have not been well described. Information about predictors of exposure are necessary to inform efforts to design epidemiological studies of PFR toxicity, prevent future exposure, and target appropriate safety information.

We conducted a pilot study among pregnant women in Rhode Island to: 1) characterize the occurrence and concentrations of urinary metabolites of PFRs, 2) evaluate the variability of urinary metabolites of these chemicals over the course of pregnancy and explore the utility of pooled urine samples for future research, and 3) investigate associations of these urinary metabolites with sociodemographic and dietary predictors.

## Methods

### Study setting and population

Between July and December 2014, we enrolled 62 women from prenatal clinics affiliated with Women & Infants Hospital of Rhode Island (WIHRI), which provides care for 75% of deliveries to state residents. Women were eligible for enrollment if they were ≥18 years old, ≤20 weeks gestation, English speaking residents of Rhode Island, and intended to deliver at WIHRI. Women were excluded if they had a multifetal pregnancy or had been diagnosed with or were currently receiving treatment for serious chronic health issues including, thyroid/renal disorders, HIV, cardiovascular disease other than hypertension, cancer, drug/alcohol addiction, or pre-gestational diabetes. Three women withdrew from the study, leaving 59 women for the present analysis. All women provided written informed consent prior to engaging in study activities and all protocols were approved by the WIHRI institutional review board. The involvement of the Centers for Disease Control and Prevention (CDC) laboratory did not constitute engagement in human subjects research.

### Urine sample collection and quantification of urinary flame retardant metabolite concentrations

We collected spot urine samples in polypropylene specimen cups during clinic visits at three time points during pregnancy: enrollment (12 ± 2 gestational weeks) and two visits coinciding with routine antenatal gestational diabetes screening (28 ± 2 gestational weeks) and group B streptococcus (35 ± 1 gestational weeks) screening tests. All 59 women provided at least one urine sample during pregnancy, 54 (91%) provided at least two samples, and 41 (70%) provided all three samples. Urine samples were immediately refrigerated following collection. Urine samples were vortexed for 30 s and specific gravity (SG) was measured using a handheld digital refractometer (ATAGO, PAS-10S) to quantify urine dilution. Then urine was aliquoted into polypropylene cryovials and stored at −80 °C within 24 h of collection. We also created a pooled urine sample for each woman using 1 mL of urine from each of her individual samples at the time of preparing the samples for shipment to the Division of Laboratory Sciences, National Center for Environmental Health, CDC (Atlanta, Georgia, USA). All samples were shipped on dry ice to the CDC, where they were stored at or below −20 °C until analysis.

Concentrations of nine urinary flame retardant metabolites, namely DPhP, BDCPP, bis-(1-chloro-2-propyl) phosphate (BCPP), bis-2-chloroethyl phosphate (BCEP), di-p-cresylphosphate (DpCP), di-o-cresylphosphate (DoCP), dibutyl phosphate (DBuP), di-benzyl-phosphate (DBzP), and 2,3,4,5-tetrabromobenzoic acid (TBBA) were quantified in individual and pooled samples [3]. These are urinary metabolites of TPhP, TDCPP, tris (1-chloro-2-propyl) phosphate, TCEP, tri-p-cresylphosphate, tri-o-cresylphosphate, TBuP, tribenzyl phosphate, and 2-ethylhexyl-2,3,4,5-tetrabromobenzoate, respectively. The method uses 0.4 mL of urine and relies on an enzymatic hydrolysis of urinary conjugates followed by automated off-line solid phase extraction with a polymeric weak anion exchange cartridge to pre-concentrate the target compounds while minimizing potential urine matrix interferences and increasing overall sensitivity and specificity. The deconjugated target analytes in the urine extract are separated on an ultra-high-performance liquid chromatography system with reversed phase chromatography, and quantified by isotope dilution-negative ion electrospray ionization tandem mass spectrometry. Spiked recoveries at four concentrations of the native analytes (2, 8, 16, 30 ng/mL) ranged from 90 to 113%, depending on the analyte. Relative standard deviations of repeated analyses of urine spiked with 1, 8 and 20 μg/L of the target analytes were <10%. The limit of detection (LOD) was estimated from 20 repeated measurements of low concentration standards after plotting the standard deviation of the measured concentration versus the standard concentration. The standard deviation at zero concentration, S_0_, was determined by the y intercept of a linear regression analysis of the above plot; LODs were calculated as three times S_0_ [26]_._ The LODs for the individual metabolites ranged from 0.05 to 0.16 μg/L, depending on the analyte (Additional file 1: Table S1). Along with the study samples, each analytical run included high- and low-concentration quality control materials (QCs) and reagent blanks to assure the accuracy and reliability of the data. The concentrations of the high-concentration QCs and the low-concentration QCs, averaged to obtain one measurement of high-concentration QC and low-concentration QC for each run, were evaluated using standard statistical probability rules [27]. In addition to the internal CDC QC procedures, we incorporated three field blanks (made with laboratory grade water) and 12 masked QC specimens from a single urine pool prepared at Brown University. The coefficients of variation (SD/mean concentration) of the blind QCs were <11% for the six analytes detected in at least 75% of samples (Additional file 1: Table S2). Concentrations of all analytes were < LOD in the reagent and field blanks.

Concentrations of urinary metabolites of interest were SG standardized using a modification of a previously described formula: *P*_*c*_ = *P*[*SG*_*ref*_-1/*SG*-1] [28], where *P*_*c*_ is the SG-standardized urinary metabolite concentration (μg/L), *P* is the concentration of the metabolite quantified in the urine sample (μg/L), *SG*_*ref*_ is the median SG within the study population at each visit (12 weeks = 1.016, 28 weeks = 1.020, 35 weeks = 1.016), and *SG* is the measured SG in each sample. For pooled samples *SG*_*ref*_ was the mean SG for all samples (1.017) and *SG* was the average SG across samples contributing to each individual’s pooled sample. For regression analyses, a log(2)-transformation was applied to urinary metabolite concentrations to decrease the influence of extreme values on effect estimates.

### Sociodemographic and dietary predictors of urinary flame retardant metabolite concentrations

At enrollment women completed a brief questionnaire describing their highest level of education attained and household income. Additional demographic (maternal age and race), anthropometric (weight and height), and perinatal factors (parity) were abstracted from the women’s medical records following delivery. Pre-pregnancy weight was available in the medical records of most women (81%). When available (n = 8), we substituted weight from the earliest prenatal care visit for women missing pre-pregnancy weight (11 gestational weeks on average; range 8–15 weeks), because weight gain in early pregnancy is generally not substantial [29]. Maternal height was available for 95% of women in the study, and body mass index (BMI) was calculated for women with both weight and height data (kg/m^2^). At approximately six weeks postpartum (mean = 6.7 ± 1.2, range = 4.4–9.1) women provided information about their food intake during pregnancy by completing the PrimeScreen, a brief dietary survey with good validity and reproducibility compared to more extensive semi-quantitative food frequency questionnaires [30].

### Statistical analysis

We examined the distributions and frequencies of participants’ sociodemographic characteristics. For flame retardant metabolites with >70% of concentrations above the LOD, we imputed concentrations < LOD with the LOD/√2 [31, 32]. Urinary metabolites that were not frequently detected (<70% detected) were not explored further (Additional file 1: Table S1). For metabolites detected in >70% of the individual samples, we report the distribution of SG-standardized concentrations in individual and pooled urine samples. Among women contributing at least two urine samples during pregnancy, we calculated intraclass correlation coefficients (ICCs) using linear mixed models with random intercepts and an unstructured covariance matrix to estimate between- and within-subject variability of log(2)-transformed urinary flame retardant metabolite concentrations over the course of pregnancy. In order to further examine changes in urinary flame retardant metabolite concentrations over the course of pregnancy, we assessed the association between repeated measurements of urinary metabolites and gestational week of urine collection using linear mixed models with unstructured covariance. We explored whether the concentrations of metabolites of interest were associated with time of urine collection (morning versus afternoon) using linear mixed models with unstructured covariance.

In order to assess whether within-subject pooling of urine might be useful for reducing potential exposure misclassification in future epidemiological studies [33], we investigated how well the urinary flame retardant metabolite concentrations measured in the pooled urine samples approximated average urinary metabolite concentrations over the course of pregnancy. We took the average concentration of each metabolite across the individual samples for each woman and calculated the ICCs comparing this arithmetic average with the measured concentration in her pooled urine sample. We also created Bland Altman plots to visualize the agreement between concentrations in pooled samples and the arithmetic average. We additionally calculated Spearman correlations among the frequently detected urinary flame retardant metabolites in the pooled samples.

We created linear mixed models with unstructured covariance to assess the association of each individual sociodemographic predictor of interest with repeated measurements of log(2)-transformed SG-standardized flame retardant metabolite concentrations; models were adjusted for gestational week of urine collection. Predictors of interest included: maternal age, pre-pregnancy weight, pre-pregnancy BMI, maternal race, education, household income, and parity. We also fit multivariable linear mixed models with unstructured covariance matrices for each PFR metabolite to estimate associations of repeated log(2)-transformed, SG-standardized, urinary concentrations with continuous age at delivery (years), pre-pregnancy weight (kg), household income (dollars), gestational week of urine collection (weeks), and indicator variables for race/ethnicity, education, and parity in the multivariable models. To further assess the utility of pooled urine samples, we created comparable multivariable linear regression models to assess the association of concentrations of PFR metabolites in the pooled urine sample with sociodemographic predictors for comparison with the results of the linear mixed models.

Multivariable linear mixed models with unstructured covariance matrices were employed to examine dietary predictors of urinary flame retardant metabolites. Each model described the association of a single dietary predictor of interest with repeated measures of log(2)-transformed SG-standardized metabolite concentrations, adjusted for continuous age at delivery (years), pre-pregnancy weight (kg), household income (dollars), gestational week at urine collection (weeks), and indicator variables for race/ethnicity, education, and parity. We included observations from 51 women with complete covariate data in the final multivariable models. For all regression models described above, we estimated percent difference in metabolite concentration [% diff = (2^ß^-1)*100], where β is the estimated regression coefficient of interest.

## Results

On average, women in our study were 29.5 years old at delivery, weighed 74.7 kg (~165 pounds) prior to pregnancy, and had a pre-pregnancy BMI of 27.7 kg/m^2^. Most participants were non-Hispanic white (59%), had a bachelor’s, graduate, or other professional degree (44%), and were parous (54%) (Table 1).

**Table 1:** Selected participant characteristics and unadjusted percent difference in urinary flame retardant metabolite concentrations as a function of sociodemographic predictors

	n	Mean ± SD	Urinary flame retardant metabolites during pregnancy^a^		
		(%)	BCEP	BDCPP	DPhP
Characteristics			% diff (95% CI)	% diff (95% CI)	% diff (95% CI)
Maternal Age (years)	52	29.5 ± 4.5	−3.2 (−10.2, 4.2)	−4.9 (−9.7, 0.1)*	−1.4 (−5.2, 2.5)
Maternal Weight (kg)	56	74.7 ± 20.0	0.9 (0.0, 1.9)*	1.3 (0.1, 2.5)**	0.5 (−0.1, 1.1)
Maternal BMI (kg/m^2^)	56	27.7 ± 6.8	1.7 (−1.5, 5.0)	3.5 (0.2, 6.9)**	1.5 (−0.2, 3.3)*
Maternal Race^b^					
Non-Hispanic White	35	(59)	0 (reference)	0 (reference)	0 (reference)
Other	20	(34)	9.1 (−34.4, 81.2)	40.5 (−10.2, 119.9)	10 (−20.7, 52.6)
Maternal Education^c^					
High School or Less	16	(27)	0 (reference)	0 (reference)	0 (reference)
Tech school/Some College	16	(27)	1.3 (−53.2, 119.6)	−40.7 (−65.7, 2.8)*	−1.1 (−35.4, 51.6)
Bachelor's/Graduate/Professional	26	(44)	−30.3 (−68.2, 52.9)	−47.1 (−67.8, −13.3)**	−4.0 (−36.2, 44.4)
			p-trend = 0.30	p-trend = 0.02	p-trend = 0.83
Household Income^c^					
<$25,000	20	(34)	0 (reference)	0 (reference)	0 (reference)
$25,000–100,000	20	(34)	−6.5 (−51.2, 79.2)	−41.3 (−64.2, −3.8)**	−6.3 (−35.1, 35.4)
>$100,000	18	(31)	−29.9 (−63.1, 33.3)	−42.8 (−64.6, −7.6)**	−10.2 (−35.8, 25.6)
			p-trend = 0.27	p-trend = 0.02	p-trend = 0.52
Parity^d^					
Nulliparous	23	(39)	0 (reference)	0 (reference)	0 (reference)
Parous	32	(54)	−1.0 (−41.5, 67.7)	31.2 (−16.6, 106.3)	1.6 (−26.3, 40.0)

*BMI* Body Mass Index, *SD* Standard deviation, *p < 0.10, **p < 0.05

^a^ Percent difference calculated from linear mixed models using the individual sociodemographic factor of interest to predict repeated measurements at 12, 28, and 35 weeks’ gestation of log(2)-transformed specific gravity standardized concentration of each urinary flame retardant metabolite adjusted for gestational week of urine sample

^b^ Missing n = 4

^c^ Missing n = 1

^d^ Missing n = 4

We frequently detected (% detected): DPhP (95%), BDCPP (93%), and BCEP (74%), and only occasionally BCPP (53%), DBuP (33%), DpCP (18%), and DoCP (1%). DBzP and TBBA were not detected in any of the urine samples (Additional file 1: Table S1). Median (IQR) BCEP, BDCPP, and DPhP, SG-standardized concentrations from pooled samples were: 0.31 μg/L (0.17–0.54), 1.18 μg/L (0.64–2.19), and 0.93 μg/L (0.72–1.97) (Additional file 1: Table S3).

**Fig. 1:**
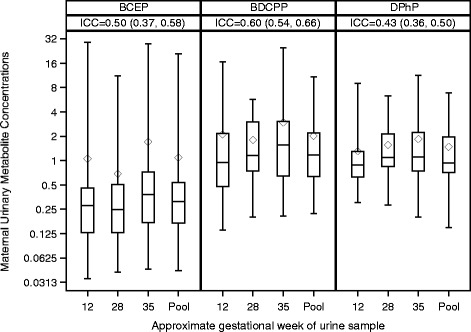
Distributions of specific gravity standardized maternal urinary replacement flame retardant metabolite concentrations (in μg/L) in urine samples from 12, 28, and 35 gestational weeks and the pooled urine sample. Intraclass correlation coefficients (ICCs) and 95% confidence intervals for the 12, 28, and 35 week samples were BCEP = 0.50, BDCPP = 0.60, and DPhP = 0.43

Applying the ICC metrics of Rosner (ICCs ≤0.4, between 0.4 and 0.75, and ≥0.75 designate poor, fair to good, and excellent reproducibility, respectively) [34], we observed fair to good reproducibility in BCEP (ICC = 0.50; 95% CI: 0.37, 0.58), BDCPP (ICC = 0.60; 95% CI: 0.54, 0.66), and DPhP (ICC = 0.43; 95% CI: 0.36, 0.50) (Fig. 1; Additional file 1: Table S4). Each additional gestational week at urine collection was marginally associated with 1.4% higher urinary BCEP (95% CI:-0.3, 3.1), 1.2% higher BDCPP (95% CI: 0.0, 2.4), and 0.9% higher DPhP (95% CI: -0.1, 1.9) (Table 2). The majority of urine samples (74%) were collected before noon. No statistically significant differences in concentrations between samples collected in the morning and afternoon were observed. However, BCEP concentrations were 44% higher in samples collected during the afternoon, though the confidence interval was wide (95% CI: -10, 131). We observed excellent agreement between the average urinary flame retardant metabolite concentrations across pregnancy samples versus concentrations in the pooled urine samples; ICC (95% CI): BCEP = 0.95 (0.91, 0.97), BDCPP = 0.89 (0.86, 0.95), and DPhP = 0.93 (0.92, 0.95) (Additional file 1: Table S5). The Bland Altman plots also supported high agreement between methods (Additional file 1: Figure S1). The SG-standardized urinary concentrations of BCEP, BDCPP, and DPhP from pooled samples were moderately positively correlated to one another with Spearman correlation coefficients ranging from 0.46 to 0.51 (*p*-values < 0.01) (Additional file 1: Table S6).

**Table 2:** Adjusted percent difference in urinary flame retardant metabolite concentrations as a function of sociodemographic predictors

	Urinary flame retardant metabolite during pregnancy^a^		
	BCEP	BDCPP	DPhP
Predictors	% diff (95% CI)	% diff (95% CI)	% diff (95% CI)
Maternal Age	−0.7 (−10, 9.6)	−3.8 (−10.9, 3.9)	−2.2 (−6.9, 2.8)
Maternal Weight (kg)	1.1 (0.1, 2.1)**	1.5 (0.3, 2.7)**	0.5 (0.0, 1.1)*
Maternal Race			
Non-Hispanic White	0 (reference)	0 (reference)	0 (reference)
Other	−23.4 (−67.4, 80)	18.1 (−31.6, 103.8)	10.4 (−31.5, 77.9)
Maternal Education			
High School or Less	0 (reference)	0 (reference)	0 (reference)
Tech school/Some College	3.9 (−52.9, 129.2)	−40.1 (−65.7, 4.5)*	−0.3 (−36.5, 56.6)
Bachelor's or more	−29.6 (−76.1, 107.3)	−43.7 (−75.5, 29.7)	8.7 (−33.0, 76.5)
	p-trend = 0.57	p-trend = 0.14	p-trend = 0.74
Household Income^b^	−0.9 (−8.0, 6.6)	3.4 (−4.8, 12.2)	1.1 (−3.6, 6.0)
Parity			
Nulliparous	0 (reference)	0 (reference)	0 (reference)
Parous	−1.7 (−42.6, 68.6)	64.1 (5.2, 155.9)**	13.6 (−21.7, 64.6)
Gestational week^c^	1.4 (−0.3, 3.1)*	1.2 (0.0, 2.4)*	0.9 (−0.1, 1.9)*

*p < 0.10, **p < 0.05

^a^ Percent difference estimated from multivariable linear mixed models using continuous age at delivery (years), pre-pregnancy weight (kg), household income (dollars), gestational week at urine collection, and indicator variables for race/ethnicity, education, and parity to predict repeated measures of log(2)-transformed specific gravity standardized concentration of a flame retardant metabolites

^b^ Estimates represent $10,000 increase in household income

^c^ Participants contributed up to three urine samples during pregnancy at 12, 28, and 35 weeks’ gestation on average

In the regression models assessing individual sociodemographic predictors adjusted for gestational week of urine collection, each 1 year increase in maternal age at delivery was marginally associated with 4.9% lower urinary BDCPP (95% CI: −9.7, 0.1). Each 1 kg increase in pre-pregnancy weight was suggestively associated with 0.9% higher BCEP (95% CI: 0.0, 1.9) and significantly associated with 1.3% higher BDCPP (95% CI: 0.1, 2.5) (Additional file 1: Figure S3); each 1 kg/m^2^ increase in pre-pregnancy BMI was associated with statistically significantly higher urinary BDCPP (3.5%; 95% CI: 0.2, 6.9), and suggestively associated with higher DPhP (1.5%; 95% CI: −0.2, 3.3). Women with more education tended to have lower BDCPP concentrations (p-trend 0.02). Likewise, women with higher household income had lower urinary concentrations of BDCPP (p-trend 0.02) (Table 1). In the regression models which simultaneously included all sociodemographic factors of interest, each 1 kg increase in pre-pregnancy weight was associated with a statistically significant increases in BCEP (1.1%, 95% CI: 0.1, 2.1) and BDCPP (1.5%, 95% CI: 0.3, 2.7), and marginally higher DPhP (0.5%, 95% CI: 0.0, 1.1). Parous women had 64.1% greater urinary BDCPP than nulliparous women (95% CI: 5.2, 155.9) (Table 2). Similar patterns of results were obtained from exploratory multivariable models including BMI rather than weight (Additional file 1: Table S7). Maternal weight remained a suggestive predictor of BCEP (1.6%; 95% CI: 0.0, 3.3) and BDCPP (1.2%; 95% CI: 0.1, 2.3) in the pooled urine samples; whereas, parity was of comparable magnitude but no longer a statistically significant predictor of urinary BDCPP concentration in pooled urine samples (Additional file 1: Table S8).

Overall, dietary factors were not predictive of greater urinary flame retardant metabolite concentrations in urine during pregnancy in multivariable regression models adjusted for relevant sociodemographic characteristics (Additional file 1: Figure S2 and Table S9). Lower urinary BCEP was associated with more frequent consumption of meat (beef/pork/lamb) in a main dish (−26.1%, 95% CI: −43.8, −2.8). Lower urinary BDCPP concentrations were observed among women who more frequently consumed leafy green vegetables (including spinach, lettuce, kale, turnip greens, and bok choy) (−19.9%, 95% CI: −33.5, −3.5) or carrots (−25.9%, 95% CI: −40.7, −7.4). More frequent consumption of citrus fruits was associated with lower urinary DPhP concentrations (−20.6%, 95% CI: −28.5, −11.8).

## Discussion

In this pilot study, we investigated patterns and predictors of urinary PFR metabolite concentrations over the course of pregnancy and assessed potential sociodemographic and dietary predictors. We found that BCEP, BDCPP, and DPhP were frequently detected in the urine of pregnant women in our study. Concentrations of these metabolites had fair to good reproducibility over the course of pregnancy. Weight and BMI were positively associated with flame retardant metabolite concentrations in urine in this cohort. Suggestive inverse associations between age, education, and household income with BDCPP were observed, and parous women had statistically significantly higher BDCPP urinary concentrations than nulliparous women. Overall, we did not observe strong associations between dietary predictors and urinary BCEP, BDCPP, or DPhP.

Both BDCPP and DPhP have been frequently detected in the urine of mothers [13, 14, 24, 25], young children [12, 13, 24], and the general population [10–12, 15–18]. Urinary concentrations of BDCPP and DPhP observed in our study were generally consistent with those previously observed among pregnant women [14, 25] (Additional file 1: Table S10). To the best of our knowledge, only one previous study (*n* = 16) reported urinary concentrations of BCEP among members of the general population in the United States; median concentrations observed in this pilot study (0.31 μg/L) were lower than in the prior study (0.63 μg/L) (Additional file 1: Table S10). Although only three of the metabolites were detected in >70% of samples, we measured a total of nine. The LODs for the other six compounds were quite comparable to the LODs of the three frequently detected flame retardant metabolites, suggesting that exposure to the precursors of these six metabolites may be limited among women in our study.

Our findings concerning temporal variability of BDCPP and DPhP are quite consistent with previous research. Hoffman et al. assessed urinary BDCPP and DPhP among eight pregnant women in North Carolina at two time points during pregnancy (18 and 28 weeks gestation) and following delivery, and reported fair to good reproducibility of both BDCPP (ICC = 0.4; 95% CI 0.2, 0.6) and DPhP (ICC = 0.4; 95% CI 0.2, 0.6) [14]. Likewise, Hoffman et al. observed excellent agreement of BDCPP (ICC = 0.81; 95% CI 0.75, 0.86) and fair agreement of DPhP (ICC = 0.51; 95% CI:0.42, 0.63) concentrations across 49 total repeated urine samples collected over a 5 day period from 11 healthy adults [8]. Meeker et al. [11] assessed urinary BDCPP and DPhP concentration among seven men in Massachusetts at nine time points over the course of a three month period, and reported fair to good reproducibility of urinary BDCPP (ICC = 0.55; 95% CI 0.31, 0.77) and poor reproducibility of DPhP (ICC = 0.35; 95% CI 0.17, 0.59). However, a study of 51 office workers in Massachusetts observed poor agreement across three measurements of urinary DPhP taken over the course of ~12 months (ICC = 0.13; 95% CI 0.02, 0.52) [19], suggesting that variability of DPhP may be greater over longer periods of observation. Our results are consistent with fair to good reproducibility of BDCPP and DPhP over the course of about six months and add information suggesting that urinary BCEP concentrations have similar variability to BDCPP and DPhP among pregnant women. In our adjusted models, we observed marginally statistically significant positive associations of gestational week of urine collection with concentrations of BCEP, BDCPP, and DPhP; whereas, Hoffman et al. observed a statistically imprecise but inverse association between gestational week of urine collection with urinary BDCPP and DPhP [25]. Although the studies conflict in terms of the direction of the association, both studies highlight the need to account for gestational week of urine collection in future epidemiologic research, particularly in studies that rely on only one urine sample from pregnancy for exposure measurement.

We observed good agreement between concentrations of BCEP, BDCPP, and DPhP measured in pooled samples from individual women and the average of concentrations from each woman’s serial urine samples. Evidence from animal studies [35–37] and human studies [38, 39] suggests that the half-lives of PFRs in the body are likely to be <24 h. In situations in which humans are exposed via multiple sources to a chemical with a short half-life in the body, exposure assessment with a single urine sample may lead to exposure misclassification and within-subject pooling of urine samples represents an efficient and cost-effective alternative to averaging the concentrations in serial samples [33]. Overall, the sociodemographic predictors of urinary PFR concentrations identified by the linear mixed models taking advantage of repeated measurements were similar to the multivariable linear regression using only the pooled sample. While we were able to collect up to 3 samples per woman over the course of pregnancy, additional samples may be necessary to realize the full potential of pooled samples to prevent exposure misclassification, but the exact amount needed will depend on the reproducibility of the repeated samples [33]. Our findings suggest that urinary flame retardant metabolites are potentially good candidates for use of within-subject pooling in future etiologic studies of the health outcomes associated with exposure to PFRs. Additionally, future studies could collect multiple urine samples during etiologically relevant periods (e.g., first trimester) and use pooling to provide a more efficient and cost-effective estimation of exposure during specific exposure windows of interest.

Prior examination of predictors of urinary BCEP, BDCPP, and DPhP among adults has been somewhat limited. Our findings generally suggest that women with higher weight or greater BMI tend to have higher concentrations of BCEP, BDCPP, and DPhP in their urine than lighter women. This is consistent with one prior study of pregnant women in which women who were overweight or obese before pregnancy tended to have higher urinary concentrations of BDCPP and DPhP than women with normal pre-pregnancy body mass index [25]. One possible explanation is that TPhP, the parent PFR of DPhP, has been used as a plasticizer (potentially as a substitute for dibutyl phthalates) in nail polish [38], and may be used as a plasticizer or phthalate substitute in other personal care products. If the relevant plasticizers are present in personal care products, this is one possible, though speculative, explanation for observing greater concentrations of urinary BCEP, BDCPP, and DPhP among heavier women, who may have greater body surface area and thus greater potential for exposure to chemicals present in personal care products. Further studies could examine use of personal care products to explore this hypothesis. Although we were unable to identify dietary predictors of PFRs in the present study, higher urinary concentrations of PFR metabolites among heavier women could also be explained by as yet undefined sources of PFR exposure in food or food packaging.

We observed a suggestive but not statistically significant inverse association between age and urinary BDCPP in the model assessing the individual influence of age; however age was not an important predictor of BDCPP in our multivariable models. Three prior studies suggest that age is inversely associated with urinary concentrations of BDCPP [8, 10, 17]. However, one larger study conducted by Hoffman et al. among pregnant women saw no association of BDCPP or DPhP with maternal age, race, or education [25]. We likewise did not observe differences in BDCPP or DPhP according to race/ethnicity, but saw some suggestion that women with higher educational attainment had lower urinary BDCPP concentrations. Sociodemographic predictors such as income, education, and race have frequently been associated with exposures to some environmental endocrine disrupting chemicals [40–43].

We speculate that the observed association of greater urinary BDCPP among parous women is at least potentially related to a higher prevalence of foam-containing infant and childhood products in the homes of pregnant women with previous children, as substantial evidence exists suggesting that PFRs are commonly found in a wide variety of foam-containing infant and baby products [2, 9, 12]. Additional research would be useful to confirm this hypothesis. In contrast, Hoffman et al. observed similar urinary BDCPP concentrations among parous and primiparous women, but urinary DPhP concentrations were elevated among parous women in their study population [25]. Collectively, our findings suggest that future etiologic studies assessing health outcomes potentially associated with exposure to TCEP, TDCPP, and TPhP would benefit from considering anthropometrics (i.e., weight), sociodemographic, and perinatal factors that may be associated with exposure.

We did not observe clear patterns between dietary factors and PFR metabolites in urine. PBDE flame retardants have commonly been found in meat, eggs, fish and seafood, animal fats and vegetable oils, milk and dairy products, and bakery products [44, 45]. Thus, the lower urinary BCEP concentrations among individuals more frequently consuming meat was unexpected. However, the persistence and bioaccumulative properties of PFRs are expected to differ from those of PBDEs [46]. Cequier et al. observed greater urinary BDCPP concentrations among children consuming more sugar and mothers consuming more cakes during the preceding 24 h [23]. The observed pattern of results in the present study may tentatively suggest that individuals eating more vegetables and fruits tend to have lower concentrations of urinary PFRs (Additional file 1: Figure S2). However, a more detailed assessment of diet, as well as direct analysis of food and food packaging to assess the presence of PFRs, may be necessary to further elucidate potential dietary sources of PFR exposure and are beyond the scope of the present pilot.

Our study has several limitations and strengths worth noting. Although this pilot study was small in size, we demonstrated that it is feasible to collect multiple urine samples over the course of pregnancy to better quantify gestational exposure to PFRs and create pooled urine samples that have excellent agreement with the average of individual measurements. While we did not directly measure SG in the pooled urine samples, the close agreement of ICCs using values that were and were not standardized for SG suggests that this was not a source of bias in our reported ICCs. We had a somewhat limited collection of covariates, but we were able to provide additional insight into several common sociodemographic factors known to influence exposure to other endocrine disrupting chemicals. Likewise, the use of an abbreviated dietary questionnaire may have prevented identification of potential dietary predictors of urinary PFRs. However, we were able to provide preliminary data to suggest that diet may not be the predominant source of exposure to PFRs. We were not able to assess pre- or post-pregnancy concentrations of urinary PFRs, thus our findings may not be generalizable to non-pregnant populations. Future research could incorporate preconception measurement of PFRs in order to better understand how the normal physiologic changes of pregnancy may influence urinary PFR concentrations.

## Conclusions

Collectively, our findings suggest that pregnant women in the United States are commonly exposed to TCEP, TDCPP, and TPhP. Urinary concentrations of BCEP, BDCPP, and DPhP are moderately variable over the course of pregnancy, such that future research may benefit from the use of within-person pooling of urine samples to reduce potential exposure misclassification. Although we did not observe clear evidence of dietary predictors of urinary PFR metabolites, future research would benefit from examining additional or more detailed potential food sources of PFRs, especially because body size was a predictor of urinary PFR metabolite concentrations in this and other cohorts. Considering the presence of these chemicals in the urine of pregnant women, increasing our understanding of possible health effects associated with exposure to PFRs during the sensitive window of pregnancy is of public health interest.

## Additional file

Additional file 1: Table S1. Parent flame retardants and limits of detection and percent detected of associated urinary metabolites. **Table S2.** Urinary concentrations of flame retardant metabolites and coefficients of variation (CV) in 12 blinded replicates. **Table S3.** Medians and selected percentiles of urinary flame retardant metabolites in urine during pregnancy. **Table S4.** Intraclass correlation coefficients of pilot study participants’ urinary flame retardant metabolite concentrations during pregnancy. **Table S5.** Intraclass correlation coefficients of average urinary flame retardant metabolite concentrations during pregnancy across pregnancy samples versus concentrations from a pooled urine sample among pilot study participants. **Table S6.** Spearman correlations among specific gravity standardized concentrations of flame retardant metabolites from the pooled urine samples. **Table S7.** Adjusted percent difference in urinary flame retardant metabolite concentrations as a function of sociodemographic predictors. **Table S8.** Adjusted percent difference in urinary flame retardant metabolite concentrations in pooled urine samples as a function of sociodemographic predictors (n=51). **Table S9.** Dietary predictors of urinary flame retardant metabolites during pregnancy. **Table S10.** Central tendency of urinary flame retardant metabolites (ng/mL) among adults in the United States. **Figure S1.** Bland Altman plots of the difference between urinary flame retardant metabolite concentrations measured in pooled urine samples and the average of concentrations from urine samples collected across pregnancy versus the mean of the two measurements. **Figure S2.** Adjusted percent difference in urinary flame retardant metabolites and 95% confidence intervals as a function of dietary factors. **Figure S3.** Scatterplots and trend lines for of log(2)transformed urinary flame retardant concentrations from pooled urine samples by maternal weight (kg). (DOCX 452 kb)

## Abbreviations

BCEP: Bis-2-chloroethyl phosphate
BCPP: Bis-(1-chloro-2-propyl) phosphate
BDCPP: Bis(1,3-dichloro-2-propyl) phosphate
BMI: Body mass index
CDC: Centers for disease control and prevention
DBuP: Dibutyl phosphate
DBzP: Di-benzyl-phosphate
DoCP: Di-o-cresylphosphate
DpCP: Di-p-cresylphosphate
DPhP: Diphenyl phosphate
ICC: Intraclass correlation coefficient
IQR: Interquartile range
LOD: Limit of detection
PFR: Organophosphate flame retardant
QC: Quality control
SG: Specific gravity
TBBA: 2,3,4,5-tetrabromobenzoic acid
TBuP: Tributyl phosphate
TCEP: Tris (2-chloroethyl) phosphate
TDCPP: Tris (1,3-dichloro-2-propyl) phosphate
TPhP: Triphenyl phosphate
US: United States
WIHRI: Women & Infants Hospital of Rhode Island
