# Supplementary material for: Variability and predictors of urinary concentrations of organophosphate flame retardant metabolites among pregnant women in Rhode Island
Source: Environ Health. 2017 Apr 11;16:40. doi: 10.1186/s12940-017-0247-z (PMC5387223; doi:10.1186/s12940-017-0247-z)
Supplement: Additional file 1: Table S1. — Parent flame retardants and limits of detection and percent detected of associated urinary metabolites. Table S2. Urinary concentrations of flame retardant metabolites and coefficients of variation (CV) in 12 blinded replicates. Table S3. Medians and selected percentiles of urinary flame retardant metabolites in urine during pregnancy. Table S4. Intraclass correlation coefficients of pilot study participants’ urinary flame retardant metabolite concentrations during pregnancy. Table S5. Intraclass correlation coefficients of average urinary flame retardant metabolite concentrations during pregnancy across pregnancy samples versus concentrations from a pooled urine sample among pilot study participants. Table S6. Spearman correlations among specific gravity standardized concentrations of flame retardant metabolites from the pooled urine samples. Table S7. Adjusted percent difference in urinary flame retardant metabolite concentrations as a function of sociodemographic predictors. Table S8. Adjusted percent difference in urinary flame retardant metabolite concentrations in pooled urine samples as a function of sociodemographic predictors (n=51). Table S9. Dietary predictors of urinary flame retardant metabolites during pregnancy. Table S10. Central tendency of urinary flame retardant metabolites (ng/mL) among adults in the United States. Figure S1. Bland Altman plots of the difference between urinary flame retardant metabolite concentrations measured in pooled urine samples and the average of concentrations from urine samples collected across pregnancy versus the mean of the two measurements. Figure S2. Adjusted percent difference in urinary flame retardant metabolites and 95% confidence intervals as a function of dietary factors. Figure S3. Scatterplots and trend lines for of log(2)transformed urinary flame retardant concentrations from pooled urine samples by maternal weight (kg). (DOCX 452 kb) [file 12940_2017_247_MOESM1_ESM.docx]

**Supplemental Material**

Variability and Predictors of Urinary Concentrations of Organophosphate Flame Retardants among Pregnant Women in Rhode Island

Megan E. Romano, Nicola Hawley, Melissa Eliot, Antonia M. Calafat, Nayana K. Jayatilaka, Karl Kelsey, Stephen McGarvey, Maureen Phipps, David Savitz, Erika .F. Werner, & Joseph M. Braun

Table of Contents:

Table S1: Parent flame retardants and limits of detection and percent detected of associated urinary metabolites ……………………………………………………………………………………………..…. 2

Table S2: Urinary concentrations of flame retardant metabolites and coefficients of variation (CV) in 12 blinded replicates …………………………………………………………………. …………….…. 3

Table S3: Medians and selected percentiles of urinary flame retardant metabolites in urine during pregnancy ..…………………………………………..………………………………………...……….…. 4

Table S4: Intraclass correlation coefficients of pilot study participants’ urinary flame retardant metabolite concentrations during pregnancy ………………………………………………………………….……… 5

Table S5: Intraclass correlation coefficients of average urinary flame retardant metabolite concentrations during pregnancy across pregnancy samples versus concentrations from a pooled urine sample among pilot study participants ……………………………………………………………………………..….….. 6

Table S6: Spearman correlations among specific gravity standardized concentrations of flame retardant metabolites from the pooled urine samples ………………….…………….………………………...…..... 7

Table S7: Adjusted percent difference in urinary flame retardant metabolite concentrations as a function of sociodemographic predictors……………………………….………………………….……………….. 8

Table S8: Adjusted percent difference in urinary flame retardant metabolite concentrations in pooled urine samples as a function of sociodemographic predictors (n=51) …………………………………...… 9

Table S9: Dietary predictors of urinary flame retardant metabolites during pregnancy ………………… 10

Table S10: Central tendency of urinary flame retardant metabolites (ng/mL) among adults in the United States …………………………………………………………………………………………………….. 11

Figure S1: : Bland Altman plots of the difference between urinary flame retardant metabolite concentrations measured in pooled urine samples and the average of concentrations from urine samples collected across pregnancy versus the mean of the two measurements ……………………………...…. 12

Figure S2: Adjusted percent difference in urinary flame retardant metabolites and 95% confidence intervals as a function of dietary factors .……………………………….……………………………...…13

Figure S3: Scatterplots and trend lines for of log(2)transformed urinary flame retardant concentrations from pooled urine samples by maternal weight (kg) ……………………………………………………. 14

Table S1: Parent flame retardants, limits of detection and detection frequency of associated urinary metabolites

|  |  |  | Detection frequency (%) | | | | |
| --- | --- | --- | --- | --- | --- | --- | --- |
| Parent Chemical | **Urinary Metabolite (Abbreviation)** | **LOD**  **(µg/L)** | **12 weeks** | **28**  **weeks** | **35**  **weeks** | **Pool** | **Overall ^a^** |
| Triphenyl phosphate | Diphenyl phosphate (DPhP) | 0.16 | 93 | 96 | 96 | 95 | 95 |
| Tris-(1,3-dichloro-2-propyl) phosphate | Bis-(1,3-dichloro-2-propyl) phosphate (BDCPP) | 0.11 | 86 | 94 | 98 | 97 | 93 |
| Tris-2-chloroethyl phosphate | Bis-2-chloroethyl phosphate (BCEP) | 0.08 | 66 | 73 | 71 | 83 | 74 |
| Tris-(1-chloro-2-propyl) phosphate | Bis-(1-chloro-2-propyl) phosphate (BCPP) | 0.10 | 55 | 49 | 57 | 54 | 53 |
| Tributyl phosphate | Dibutyl phosphate (DBuP) | 0.05 | 39 | 27 | 29 | 34 | 33 |
| Tri-p-cresylphosphate | Di-p-cresylphosphate (DpCP) | 0.05 | 16 | 20 | 18 | 19 | 18 |
| Tri-o-cresylphosphate | Di-o-cresylphosphate (DoCP) | 0.05 | 2 | 2 | 0 | 0 | 1 |
| Tri-benzyl-phosphate | Di-benzyl-phosphate (DBzP) | 0.05 | 0 | 0 | 0 | 0 | 0 |
| 2-ethylhexyl-2,3,4,5-tetrabromobenzoate | 2,3,4,5-tetrabromobenzoic acid (TBBA) | 0.05 | 0 | 0 | 0 | 0 | 0 |

^a^ Detection frequencies across all samples measured regardless of timing

Table S2: Urinary concentrations of flame retardant metabolites and coefficients of variation (CV) in 12 blinded replicates

| Analyte | n detected | Median | Min | Max | Mean±SD | CV |
| --- | --- | --- | --- | --- | --- | --- |
| Dibutyl phosphate (DBuP) | 9 | 0.17 | 0.16 | 0.21 | 0.17±0.02 | 10.2% |
| Bis-2-chloroethyl phosphate (BCEP) | 12 | 12.0 | 10.5 | 13.6 | 11.9±0.9 | 7.9% |
| Bis-(1,3-dichloro-2-propyl) phosphate (BDCPP) | 12 | 7.4 | 6.4 | 8.2 | 7.2±0.6 | 7.6% |
| Diphenyl phosphate (DPhP) | 12 | 20.5 | 18.9 | 23.3 | 20.8±1.4 | 6.7% |
| Bis-(1-chloro-2-propyl) phosphate (BCPP) | 12 | 10.7 | 9.9 | 11.8 | 10.8±0.7 | 6.2% |
| Di-p-cresylphosphate (DpCP) | 12 | 0.71 | 0.64 | 0.78 | 0.71±0.04 | 5.5% |
| Di-o-cresylphosphate (DoCP) | 0 |  |  |  |  |  |
| Di-benzyl-phosphate (DBzP) | 0 |  |  |  |  |  |
| 2,3,4,5-tetrabromobenzoic acid (TBBA) | 0 |  |  |  |  |  |

Table S3: Medians and select percentiles (in µg/L) of urinary flame retardant metabolite concentrations in urine during pregnancy

|  | 12 weeks ( n=56) | 28 weeks (n=49) | 35 weeks (n=47) | Average (n=59)^a^ | Pool (n=58)^b^ |
| --- | --- | --- | --- | --- | --- |
|  | Median (25^th^-75^th^) | Median (25^th^-75^th^) | Median (25^th^-75^th^) | Median (25^th^-75^th^) | Median (25^th^-75^th^) |
| BCEP |  |  |  |  |  |
| Unstandardized | 0.20 (0.06-0.56) | 0.24 (0.06-0.49) | 0.33 (0.06-0.64) | 0.31 (0.11-0.80) | 0.35 (0.11-0.51) |
| SG-standardized | 0.28 (0.13-0.46) | 0.25 (0.13-0.51) | 0.38 (0.17-0.72) | 0.32 (0.19-0.64) | 0.31 (0.17-0.54) |
| BDCPP |  |  |  |  |  |
| Unstandardized | 1.09 (0.35-2.83) | 1.39 (0.64-3.42) | 1.31 (0.59-3.36) | 1.43 (0.62-3.06) | 1.38 (0.45-2.43) |
| SG-standardized | 0.94 (0.48-2.16) | 1.16 (0.74-2.99) | 1.55 (0.65-3.04) | 1.40 (0.60-2.65) | 1.18 (0.64-2.19) |
| DPhP |  |  |  |  |  |
| Unstandardized | 0.86 (0.40-1.71) | 1.06 (0.60-2.17) | 1.13 (0.53-2.51) | 1.11 (0.59-2.17) | 1.02 (0.52-1.94) |
| SG-standardized | 0.89 (0.63-1.29) | 1.10 (0.84-2.12) | 1.11 (0.75-2.24) | 1.24 (0.76-1.93) | 0.93 (0.72-1.97) |

^a^ Includes all women that contributed at least one urine sample over the course of pregnancy ^b^ Excludes one sample for which a pooling error was identified after laboratory analysis

Table S4: Intraclass correlation coefficients of pilot study participants’ urinary flame retardant metabolite concentrations during pregnancy

|  | **Pregnancy ICCs ^a^** | | | | | |
| --- | --- | --- | --- | --- | --- | --- |
|  | Unstandardized ^b^ | | | SG Standardized ^c^ | | |
| **Metabolite** | ICC (95% CI) | Between Subject Variance | Within Subject Variance | ICC (95% CI) | Between Subject Variance | Within Subject Variance |
| **BCEP** | 0.57 (0.47, 0.65) | 2.70 | 2.03 | 0.50 (0.37, 0.58) | 1.76 | 1.76 |
| **BDCPP** | 0.61 (0.56, 0.67) | 2.25 | 1.41 | 0.60 (0.54, 0.66) | 1.27 | 0.86 |
| **DPhP** | 0.51 (0.45, 0.58) | 1.22 | 1.15 | 0.43 (0.36, 0.50) | 0.49 | 0.65 |

^a^ Intraclass correlation coefficients for the urine samples from ~12, ~28, and ~35 gestational weeks among subjects with at least 2 urine samples( n=54)

^b^ Intraclass correlation coefficients for urinary flame retardant metabolites without standardization for urine specific gravity

^c^ Intraclass correlation coefficients for urinary flame retardant metabolites with standardization for urine specific gravity

Table S5: Intraclass correlation coefficients of average urinary flame retardant metabolite concentrations during pregnancy across pregnancy samples versus concentrations from a pooled urine sample among pilot study participants

| **Pregnancy average versus pooled sample ICCs^a^** | | | | | | |
| --- | --- | --- | --- | --- | --- | --- |
|  | Unstandardized ^b^ | | | SG Standardized ^c^ | | |
| Analyte | ICC (95% CI) | Between Subject Variance | Within Subject Variance | ICC (95% CI) | Between Subject Variance | Within Subject Variance |
| BCEP | 0.96 (0.94, 0.98) | 4.16 | 0.17 | 0.95 (0.91, 0.97) | 3.01 | 0.17 |
| BDCPP | 0.92 (0.90, 0.97) | 2.61 | 0.21 | 0.89 (0.86, 0.95) | 1.54 | 0.19 |
| DPhP | 0.97 (0.96, 0.98) | 1.86 | 0.05 | 0.93 (0.92, 0.95) | 0.95 | 0.07 |

^a^ Intraclass correlation coefficients for the average concentration of urinary metabolites in samples from ~12, ~28, and ~35 gestational weeks versus concentration in pooled sample among subjects with at least 2 urine samples( n=54)

^b^ Intraclass correlation coefficients for urinary flame retardant metabolites without standardization for urine specific gravity

^c^ Intraclass correlation coefficients for urinary flame retardant metabolites with standardization for urine specific gravity

Table S6: Spearman correlations among specific gravity standardized concentrations of flame retardant metabolites from the pooled urine samples

| Spearman correlation coefficient (p-value) | | |
| --- | --- | --- |
| Analyte | BCEP | BDCPP |
| BDCPP | 0.47 (0.0003) | -- |
| DPhP | 0.51 (<0.0001) | 0.46 (0.0004) |

Table S7: Adjusted percent difference in urinary flame retardant metabolite concentrations as a function of sociodemographic predictors

|  | Urinary flame retardant metabolite during pregnancy ^a^ | | | | | |
| --- | --- | --- | --- | --- | --- | --- |
|  | BCEP | | BDCPP | | DPhP | |
| Predictors | % diff (95% CI) |  | % diff (95% CI) |  | % diff (95% CI) |  |
| Maternal Age | -0.9 (-10.5, 9.7) |  | -4.1 (-11.3, 3.7) |  | -2.2 (-6.9, 2.7) |  |
| Maternal Body Mass Index (kg/m^2^) | 1.9 (-1.3, 5.3) |  | 3.7 (0.4, 7.0) | ** | 1.8 (0.1, 3.6) | ** |
| Maternal Race |  |  |  |  |  |  |
| Non-Hispanic White | 0 (reference) |  | 0 (reference) |  | 0 (reference) |  |
| Other | -24.8 (-68.9, 81.6) |  | 14.0 (-34.9, 99.5) |  | 8.4 (-32.6, 74.3) |  |
| Maternal Education |  |  |  |  |  |  |
| High School or Less | 0 (reference) |  | 0 (reference) |  | 0 (reference) |  |
| Tech school/Some College | 10.4 (-50.3, 145.2) |  | -36.1 (-63.8, 13.0) |  | 1.4 (-35.9, 60.4) |  |
| Bachelor's or more | -27.8 (-76, 117.5) |  | -43.3 (-75.1, 29.4) |  | 8.4 (-33.1, 75.4) |  |
|  | p-trend=0.63 |  | p-trend=0.15 |  | p-trend=0.75 |  |
| Household Income ^b^ | -1.1 (-8.2, 6.6) |  | 3.7 (-4.2, 12.2) |  | 1.4 (-3.2, 6.2) |  |
| Parity |  |  |  |  |  |  |
| Nulliparous | 0 (reference) |  | 0 (reference) |  | 0 (reference) |  |
| Parous | -3.5 (-44.3, 67.1) |  | 59.6 (0.1, 154.3) | ** | 13 (-21.6, 62.7) |  |
| Gestational week ^c^ | 1.4 (-0.3, 3.1) |  | 1.2 (0, 2.4) | * | 0.9 (-0.1, 1.9) | * |

*p<0.10, **p<0.05 ^a^ Percent difference estimated from multivariable linear mixed models using continuous age at delivery (years), pre-pregnancy body mass index (kg/m^2^), household income (dollars), gestational week at urine collection, and indicator variables for race/ethnicity, education, and parity to predict repeated measures of log(2)-transformed specific gravity standardized concentration of a flame retardant metabolites ^b^ Estimates represent $10,000 increase in household income ^c^ Participants contributed up to three urine samples during pregnancy at 12, 28, and 35 weeks’ gestation on average

Table S8: Adjusted percent difference in urinary flame retardant metabolite concentrations in pooled urine samples as a function of sociodemographic predictors (n=51)

|  | Urinary flame retardant metabolite during pregnancy ^a^ | | | | |
| --- | --- | --- | --- | --- | --- |
|  | BCEP |  | BDCPP |  | DPhP |
| Predictors | %diff (95% CI) |  | %diff (95% CI) |  | %diff (95% CI) |
| Maternal Age | -1.2 (-11.1, 9.8) |  | -2.6 (-9.4, 4.7) |  | -2.0 (-8.5, 5.0) |
| Maternal Weight (kg) | 1.6 (0, 3.3) | * | 1.2 (0.1, 2.3) | ** | 0.5 (-0.5, 1.6) |
| Maternal Race |  |  |  |  |  |
| Non-Hispanic White | 0 (reference) |  | 0 (reference) |  | 0 (reference) |
| Other | -21.9 (-66.1, 79.7) |  | 31.6 (-25.7, 133.2) |  | 7.7 (-37.4, 85.4) |
| Maternal Education |  |  |  |  |  |
| High School or Less | 0 (reference) |  | 0 (reference) |  | 0 (reference) |
| Tech school/Some College | -20.3 (-67.9, 97.8) |  | -44.9 (-70.5, 2.7) | * | -1.5 (-45.5, 78) |
| Bachelor's or more | -32.5 (-78.1, 108.6) |  | -48.6 (-76.3, 11.3) | * | 24.4 (-40.3, 159.2) |
|  | p-trend=0.49 |  | p-trend=0.07 | * | p-trend=0.59 |
| Household Income ^b^ | 0.8 (-9.6, 12.4) |  | 2.4 (-4.9, 10.4) |  | 0.5 (-6.4, 7.9) |
| Parity |  |  |  |  |  |
| Nulliparous | 0 (reference) |  | 0 (reference) |  | 0 (reference) |
| Parous | 11.8 (-46.8, 135) |  | 44 (-13.5, 139.6) |  | 8.7 (-33, 76.3) |
|  |  | | |  |  |

*p<0.10, **p<0.05 ^a^ Percent difference estimated from multivariable linear regression models using continuous age at delivery (years), pre-pregnancy weight (kg), household income (dollars), and indicator variables for race/ethnicity, education, and parity to predict log(2)-transformed specific gravity standardized concentration of a flame retardant metabolite in the pooled urine with participants contributing up to three urine samples during pregnancy at 12, 28, and 35 weeks’ gestation on average to create the pooled sample ^b^ Estimates represent $10,000 increase in household income

Table S9: Dietary predictors of urinary flame retardant metabolites during pregnancy

|  | Urinary flame retardant metabolites during pregnancy ^a^ | | | | | |
| --- | --- | --- | --- | --- | --- | --- |
| Food Item | BCEP |  | BDCPP |  | DPhP |  |
|  | % diff (p-value) |  | % diff (p-value) |  | % diff (p-value) |  |
| Leafy greens | -21.5 (-40.1, 3) | * | -19.9 (-33.5, -3.5) | ** | -2.7 (-17.6, 14.9) |  |
| Cruciferous vegetables | -0.6 (-24.5, 30.8) |  | -14.2 (-29.7, 4.6) |  | -6.9 (-21.4, 10.3) |  |
| Carrots | -24.5 (-44.3, 2.5) | * | -25.9 (-40.7, -7.4) | ** | -15.5 (-28.7, 0.2) |  |
| Other vegetables | -21 (-42.6, 8.9) |  | -18.2 (-33.5, 0.6) | * | -1.1 (-21.2, 24) |  |
| Citrus | -18.9 (-34.4, 0.3) | * | -10.6 (-24.1, 5.3) |  | -20.6 (-28.5, -11.8) | ** |
| Other fruit | -20.9 (-45.3, 14.3) |  | -13.3 (-29.4, 6.4) |  | -5.8 (-24.1, 17) |  |
| Whole dairy | -4.6 (-22.6, 17.7) |  | -2.2 (-17.6, 16.1) |  | 1.8 (-9.2, 14.3) |  |
| Low fat dairy | -11 (-26.4, 7.7) |  | -10.8 (-25.1, 6.1) |  | -4.9 (-16.7, 8.4) |  |
| Eggs | -7.1 (-31.4, 25.6) |  | -20 (-36.4, 0.7) | * | 1.2 (-19.2, 26.8) |  |
| Margarine | 22.3 (-4.2, 56) |  | -13.6 (-29.4, 5.7) |  | 2.6 (-12.6, 20.3) |  |
| Whole grains | 7 (-19.9, 43) |  | -10.5 (-28.3, 11.7) |  | 5.4 (-11.9, 26) |  |
| Pasta, rice, noodles | -8.7 (-35.4, 29.1) |  | -13.5 (-31.8, 9.6) |  | 3.6 (-11.7, 21.4) |  |
| Baked goods | -12.4 (-35.9, 19.8) |  | 6.5 (-14.4, 32.5) |  | 0.5 (-17.3, 22.2) |  |
| Beef, pork, lamb | -26.1 (-43.8, -2.8) | ** | -13.4 (-28.9, 5.5) |  | -10.6 (-24, 5.2) |  |
| Processed meat | -4.6 (-33.6, 37) |  | 2.7 (-23.4, 37.7) |  | -7.5 (-25.8, 15.3) |  |
| Fish or seafood | -3 (-31.8, 37.9) |  | -6.9 (-29.7, 23.3) |  | -9.9 (-27.8, 12.6) |  |
| Deep fried foods | 18.1 (-18.9, 71.8) |  | 12.3 (-12.4, 44.1) |  | 3.7 (-16.4, 28.7) |  |
| Adding salt to food | 2.3 (-15, 23.1) |  | -10.5 (-24.6, 6.4) |  | 8.7 (-2.6, 21.4) |  |

*p<0.10, **p<0.05

^a^ Percent difference estimated from multivariable linear regression models using log(2)-transformed specific gravity standardized concentration of a flame retardant metabolite from pooled urine sample as outcome and adjusted for pre-pregnancy weight (kg), maternal age at delivery, household income, parity, and gestational week at urine sample collection.

Table S10: Central tendency of urinary flame retardant metabolites (µg/L) among adults in the United States

| Study | Measurement | BCEP | BDCPP | DPhP | Study location  population (n) | SG-standardized |
| --- | --- | --- | --- | --- | --- | --- |
| Present Study | Median  (25th-75th) | 0.31  (0.17-0.54) | 1.18  (0.64-2.19) | 0.93  (0.72-1.97) | Rhode Island  Pregnant women (58 pooled samples) | Yes |
| Butt et al. 2014 | GM  (95% CI) | na | 2.4  (1.5-3.7) | 1.9  (1.1-3.4) | New Jersey  Mothers of toddlers (22) | Yes |
| Butt et al. 2016 | GM | na | 3.3 | 1.2 | California  Mothers of toddlers (28) | Yes |
| Carignan et al. 2013 | GM  (95% CI) | na | 0.41  (0.28–0.59) | na | Massachusetts  Office workers (29) | Yes |
| Carignan et al. 2016 | GM  (95% CI) | na | 0.67  (0.52–0.85) | 9.0  (7.7–11.1) | United States  Collegiate gymnasts (11 women; 54 samples) | Yes |
| Cooper et al. 2011 | GM | na | 0.41 | 2.97 | North America  General population volunteers (9) | Yes |
| Dodson et al. 2014 | Median | 0.63 | 0.09 | 0.44 | California  Nonsmoking adults (16) | No |
| Hoffman et al. 2014 | Median  (25th-75th) | na | 1.1  (0.8, 2.7) | 1.6  (0.9-3.5) | North Carolina  Pregnant women (8 women; 39 samples) | No |
| Hoffman et al. 2015 | GM | na | 0.37 | 1.02 | North Carolina  Healthy adult volunteers (53) | No |
| Hoffman et al. 2017 | GM | na | 1.8 | 1.4 | North Carolina  Pregnant women (349) | Yes |
| Meeker et al. 2013 | Median  (25th-75th) | na | 0.12  (0.03-0.27) | 0.27  (0.14-0.75) | Massachusetts  Men (7 men; 106 samples) | Yes |
| Preston et al. 2017 | GM  (GSD) | na | na | 2.36  (2.30) | Massachusetts  Office workers (n=51; 135 samples) | Yes |

| 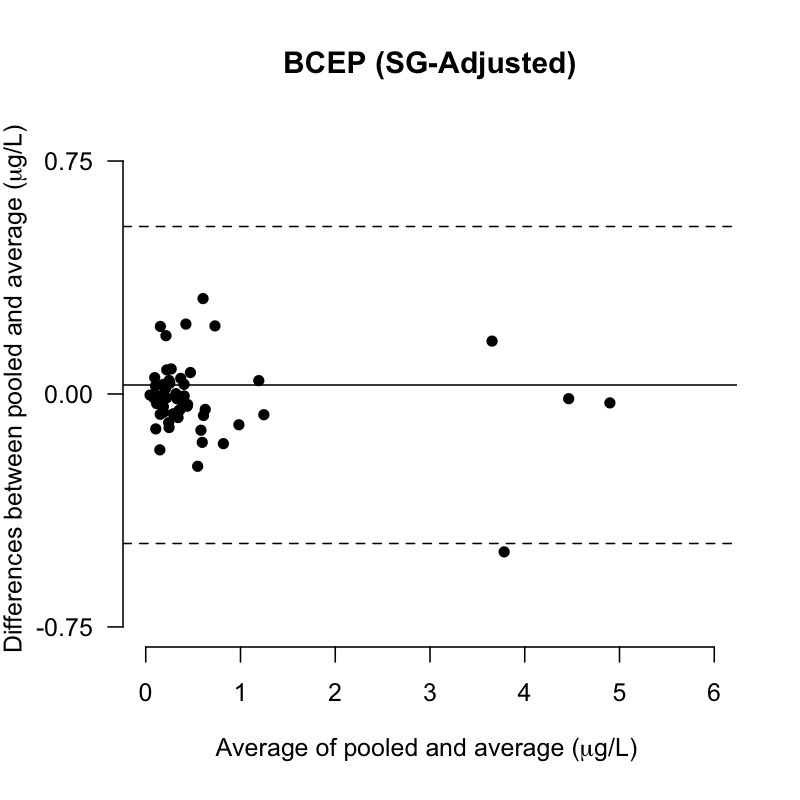 | 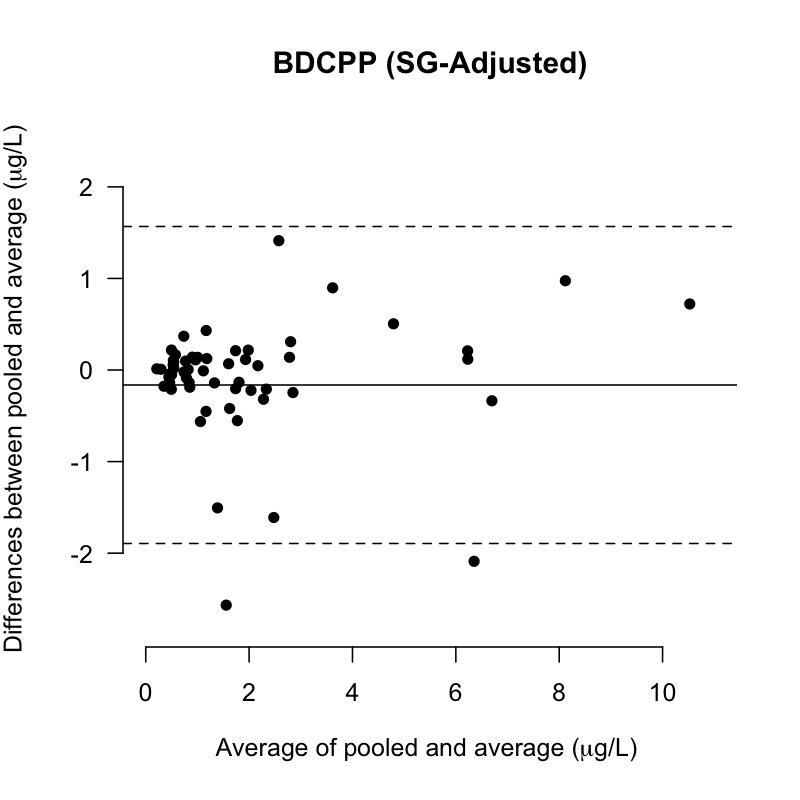 | 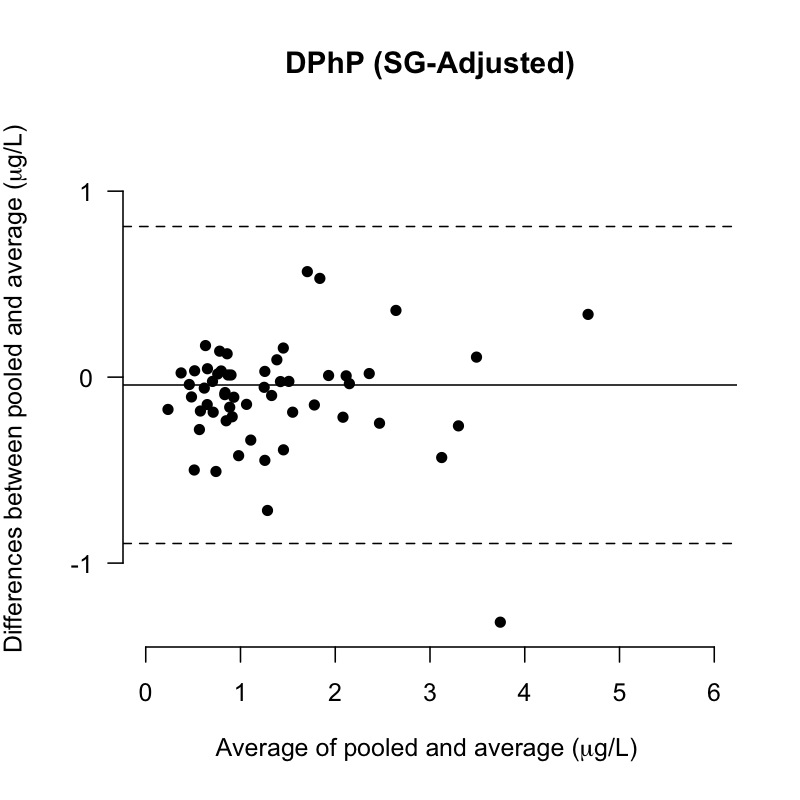 |
| --- | --- | --- |
| 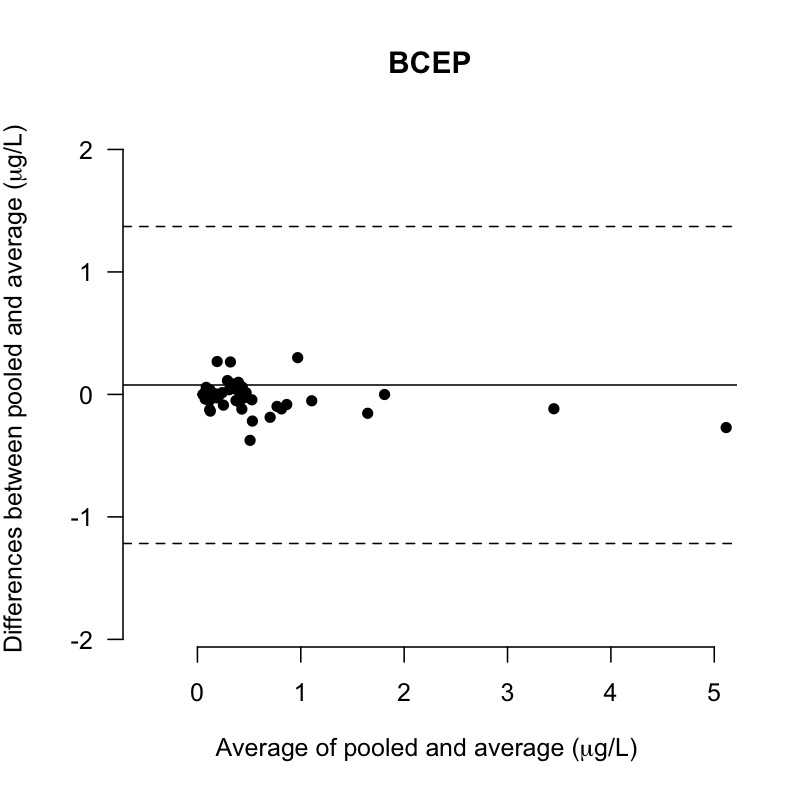 | 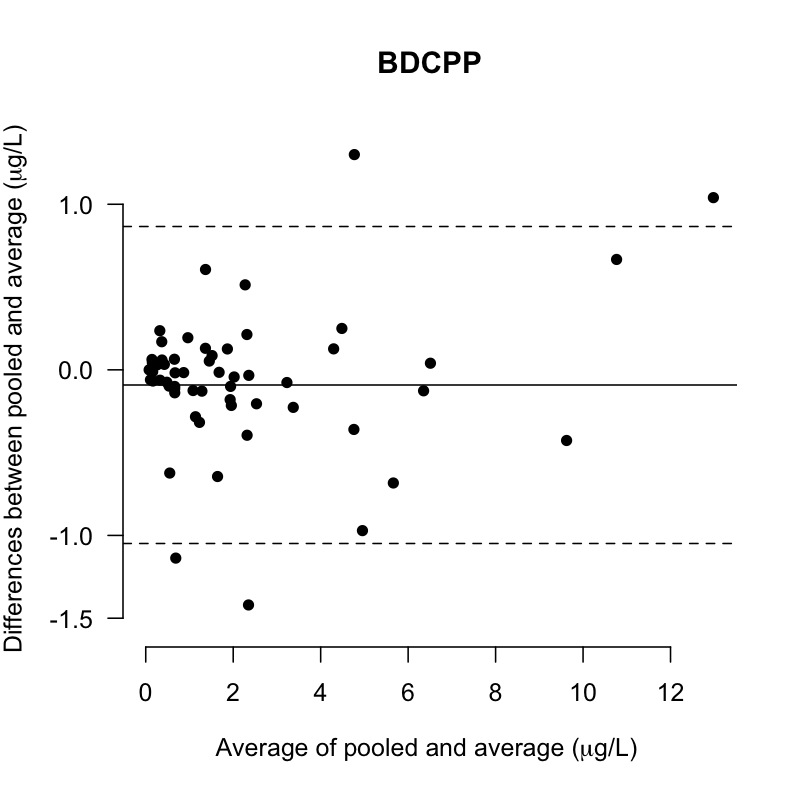 | 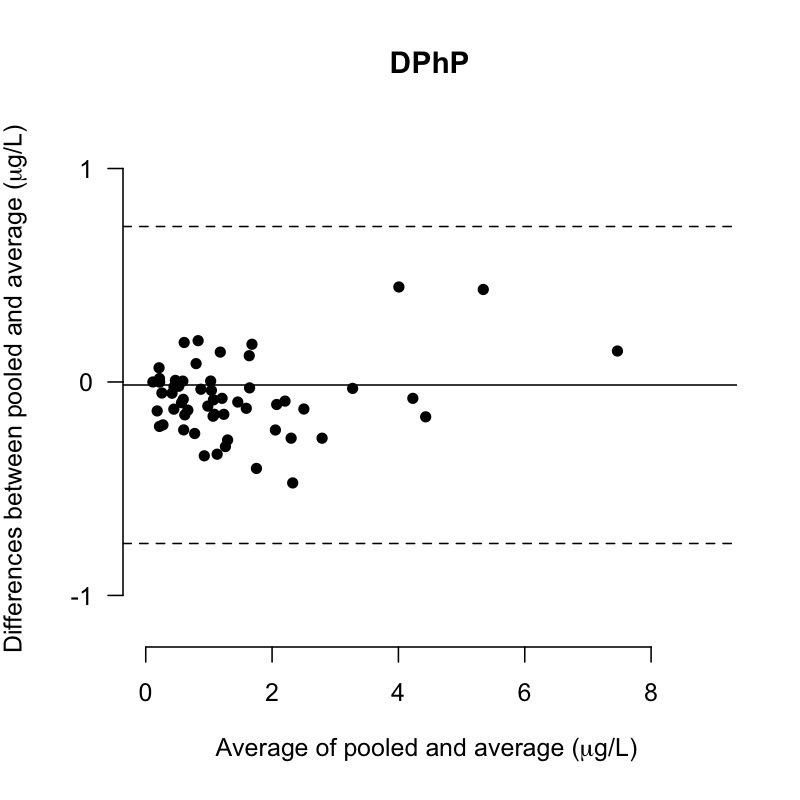 |

Figure S1: Bland Altman plots of the difference between urinary flame retardant metabolite concentrations measured in pooled urine samples and the average of concentrations from urine samples collected across pregnancy versus the mean of the two measurements

Figure S2: Adjusted percent difference in urinary flame retardant metabolites and 95% confidence intervals as a function of dietary factors. Dietary patterns during pregnancy were assessed by the PrimeScreen (Rifas-Shiman et al. 2001) and individual dietary predictors of log(2)transformed urinary flame retardant concentration from pooled urine samples were added to multivariable linear regression models including sociodemographic factors. All models were adjusted for pre-pregnancy weight (kg), maternal age at delivery, household income, parity, and gestational week at urine sample collection.

Figure S3: Scatterplots and trend lines for of log(2)transformed urinary flame retardant concentrations from pooled urine samples by maternal weight (kg)
